# Effects of plasma-derived exosomes from the normal and thin Bactrian camels on hepatocellular carcinoma and their differences at transcriptome and proteomics levels

**DOI:** 10.3389/fonc.2023.994340

**Published:** 2023-02-02

**Authors:** Hongqiang Yao, Siriguleng Yu, Yuchen Luo, Ming Wang, Xiuying Wang, Siriguleng Xu, Yufei Chen, Zhifeng Xie

**Affiliations:** ^1^ Key Laboratory of Clinical Diagnosis and Treatment Technology for Animal Diseases, Ministry of Agriculture, College of Veterinary Medicine, Inner Mongolia Agricultural University, Hohhot, Inner Mongolia, China; ^2^ Department of Public Health, Inner Mongolia Center for Disease Control and Prevention, Hohhot, Inner Mongolia, China

**Keywords:** Bactrian camel, exosomes, hepatocellular carcinoma, small sequencing, proteomics

## Abstract

**Background:**

Hepatocellular carcinoma (HCC) is a common malignant primary tumor. Bactrian camels have high economic and social values, but their potential medical value has not been studied. This study aimed to investigate the effects of Bactrian camel plasma-derived exosomes on HCC.

**Methods:**

Plasma was obtained from thin and normal Bactrian camels, and used to isolate exosomes by ultracentrifugation. The exosomes were then characterized by transmission electron microscopy and Nano particle tracking analyzer. *In vivo* imaging of nude mice and hematoxylin eosin (HE) staining of liver tissues were used to explore the effects of the exosomes on tumor growth. Finally, the differences of the two exosomes were further analyzed using small RNA sequencing and proteomics.

**Results:**

*In vivo* imaging and HE staining showed that no significant differences were found in fluorescence value and liver tissue morphology between the control mice and the mice treated with the exosomes from thin Bactrian camels; while the fluorescence value and the live histology changes were alleviated in the mice with the exosomes from normal Bactrian camels. After sequencing and proteomic analysis, 40 differentially expressed miRNAs (DE-miRNAs, 15 down-regulated and 25 up-regulated) and 172 differentially expressed proteins (DEPs, 77 up-regulated and 95 down-regulated) were identified in the plasma-derived exosomes from normal Bactrian camels. These identified DE-miRNAs and DEPs were significantly enriched in many signaling pathways.

**Conclusions:**

Normal Bactrian camel plasma-derived exosomes may inhibit the growth of HCC cells through regulating pathways of Ras, Ras-Association Proximate 1 (Rap1), phosphoinositide 3-kinase-protein kinase B (PI3K-Akt), mitogen-activated protein kinase (MAPK), adenosine monophosphate-activated protein kinase (AMPK), and canonical Wnt signaling pathways.

## Introduction

Hepatocellular carcinoma (HCC) is a common malignant primary tumor, and the second leading cause of cancer-related deaths worldwide, particular in East Asia (China, Japan, and Korea) and sub-Saharan Africa (1). Like any other cancer, the pathogenesis of HCC is complex, and there were no obvious symptoms, so most HCC patients can only be diagnosed in a late stage (2). Despite recent advances in clinical diagnosis and treatment, advanced diagnosis and metastasis remain the major causes of high HCC mortality (3). Chemotherapy is also commonly used to treat HCC, but currently used chemotherapy lacks selective targeting, and may damage normal (healthy) cells, thereby leading to serious adverse reactions (4). Its long-term use can contribute to drug resistance, which is detrimental to the prognosis and survival of HCC patients (5). In addition, the liver is a robust immunosuppressive microenvironment, characterized by high levels of immunosuppressive cytokines (IL-10, and TGF-β) released by LSEC and Treg cells, and well expression of immune checkpoint molecules (PD-1, CTLA-4, LAG-3, and TIM-3) on immune cells (6). Nowadays, immune checkpoint inhibitors (ICIs), such as tyrosine-based protein kinase inhibitors and histone deacetylase inhibitors, are used for targeted therapy of HCC (7). However, due to the drug resistance and side effects, the prognosis of HCC patients remains frustrating, with a 5-year survival rate of ~12.5% (6). Therefore, there is an urgent need to develop a more safe, effective, and selective anti-cancer drug adjuvant that can be used alone or in combination with chemotherapy or ICIs to enhance its therapeutic potential and reduce side effects.

Exosomes are about 40-160 nm extracellular vesicles that produced by different types of cells (8). Exosomes, as carriers of protein, lipid, nucleic acids (mRNA, microRNA [miRNA] and DNA) and metabolites, are mediators of near- and long-distance cell-to-cell communication, and influence various cell biological processes, including cell growth, migration, invasion and apoptosis (9). It has also been reported that exosomes play immune-activation and immunosuppression functions in cancer. The activation of immunity depends on the antigen presentation of exosomes, while the immunosuppressive effect of exosomes depends on the carried ligands, proteins, and miRNAs to suppress the activity of cytotoxic T cells or increase the immunosuppressive cells (9). Increasing evidence showed that mesenchymal stem cell-derived exosomes can promote angiogenesis, tissue regeneration and immune regulation through regulating intercellular micro-communication and transfer of paracrine factors, thus being used in the treatment of liver diseases (10). A study of Lou et al. demonstrated that exosomes from adipose tissue-derive MSCs could be an effective vector for miR-199a delivery, and effectively improve the sensitivity of HCC to chemotherapy drugs *via* targeting the mTOR pathway (11). Additionally, a previous study has reported that camel milk-derived exosomes could induce cell apoptosis and inhibit inflammation, oxidative stress, angiogenesis, and metastasis in tumor microenvironment, thus promoting the death of MCF7 cells and suppressing the progression of breast cancer (12). Another study also showed that camel milk-derived exosomes have a selective anti-proliferation effect on HepaRG cells (liver cancer cells), but have no significant cytotoxic effect on ThLE-2 cells (normal liver cells), suggesting a potential therapeutic role in cancer treatment and prevention (13). Based on these reports, we speculate that exosomes from camel or camel milk may be useful for cancer management. Furthermore, the degree of individual weight can change the contents of exosomes, such as mRNAs, miRNAs, proteins, and adipokines, thereby playing different mechanisms in the diseases (14, 15). However, the roles of exosomes from camel of different body weights in HCC remain unclear.

Bactrian camels (*Camelus bactrianus*) are widely distributed in Xinjiang, Gansu, and Inner Mongolia of China, and have unique biological characteristics, including strong thirst tolerance, hunger tolerance, and adaptability to harsh climate (16). They can store energy in the form of fat deposit, and can survive for a long time in the case of water and food shortage. The blood sugar levels of camels are more than twice as high as those of other ruminants, but they do not develop metabolic diseases and show associated pathological features (16, 17). It has also been reported that Bactrian camels may have potential immunotherapeutic roles in cancer treatment and prevention because of their unique habits and extreme living conditions (18–20). However, understanding of their potential medical values is still limited. In addition, milk is different from other body fluids (such as plasma), as well as its composition is complex, and contains a mass of small molecules of milk proteins and milk fat (21, 22). Some of the small molecules are similar in size to exosomes, which may lead to low concentration and purity of milk-derived exosomes after isolation (22, 23). To date, there is no recognized separation method that can obtain high purity, concentration, and bioactivity of milk-derived exosomes (24). Therefore, in our study, the exosomes were isolated from the plasma from the thin and normal Bactrian camels, and then their roles in HCC progression were explored. Due to the different effects of the two exosomes on HCC, the two exosomes were further analyzed using small RNA sequencing and metabolomics analyses. Our work will provide new evidence and insights into the prevention and treatment of HCC.

## Materials and methods

### Plasma sample collection

On December, 15th, 2021, thin Bactrian camels (n = 5) and normal Bactrian camels (n = 5) were obtained from Shibatai Township, Zhuozishan Town, Wulanchabu, Inner Mongolia, and the blood (80 mL) were taken from each Bactrian camel. The information (age, girth of abdomen, height, and body length) of all the Bactrian camels was shown in **Table 1**. The research protocols were approved by the Experimental Animal Welfare and Ethics Committee of Inner Mongolia Agricultural University (No. NND2021094) on November 2, 2021.

**Table 1:** Detailed characteristics of two groups camels in this study.

Sample No.			Thin Bactrian camels			Normal Bactrian camels				
	Age (years)	Weight (kg)	Girth of abdomen (cm)	Height (cm)	Body length (cm)	Age (years)	Weight (kg)	Girth of abdomen (cm)	Height (cm)	Body length (cm)
**1**	10	430	207.3	173.8	168.7	11	745	229.4	178.2	172.2
**2**	10	436	209.8	175.5	169.4	9	710	226.6	171.5	166.5
**3**	12	447	212.5	178.7	174.1	12	748	234.9	176.7	171.3
**4**	11	449	213.6	180.9	176.2	10	730	228.5	173.9	168.5
**5**	11	438	210.1	176.1	170.5	12	750	236.4	181.1	175.8

### Isolation and characterization of exosomes

The exosomes were isolated from the Bactrian camel plasma samples (n = 5 for each group) at 4°C as described previously (25) with some modifications. Briefly, the blood samples were diluted with the isopycnic PBS, and then centrifuged at 300 g for 10 min. After that, the supernatant was transferred to a new tube, and then centrifuged at 2000 g for 10 min, followed by 12000 g for 30 min, and 120000 g for 70 min. The sediments were resuspended with pre-cooling PBS (2500 μL), and the exosomes were obtained and stored at -80°C. The concentrations of the isolated exosomes were quantified using a BCA protein assay kit (Boster Biological Technology Co. Ltd, Wuhan, China) based on the manufacturer’s instructions.

Thereafter, the isolated solution (50 μL) was added to PBS (10 mL), and centrifuged at 120000 g for 70 min at 4°C. Then, the sediments were resuspended with 50 μL PBS, and used for transmission electron microscopy (TEM) and Nano particle tracking analyzer (NTA). A TEM (Tecnai G2 spititi, FEI company, OR, USA) was used to visualize the morphology and ultrastructure of the extracted exosomes based on the previous study (26). Besides, the exosomes size distribution was evaluated using a ZetaView^®^ NTA (ZetaView Particle Metrix, Particle Metrix, Germany) in accordance with the method of Soares Matins et al. (27).

### Total RNA extraction and small RNA sequencing

Total RNA was extracted from all the plasma-derived exosomes of thin and normal Bactrian camels using mirVANA miRNA Isolation kit (Takara Biomedical Technology Co., Ltd., Beijing, China) according to the manufacturer’s recommendations, and then the quality and concentrations of the total RNA were assessed using a microplate reader (OD260/280). After that, the total RNA (n = 5 for each group) was sent to OBiO Technology (Shanghai) Corp., Ltd (China) for small RNA sequencing.

During sequencing, TruSeq small RNA Sample Prep Kit (Illumina, San Diego, USA) was used to construct miRNA library. Thereafter, RNA 3’ and 5’ adaptors were ligated, as well as the enrichment library was amplified by reverse transcription-polymerase chain reaction (RT-PCR), and was purified by gel electrophoresis. After the library preparation, Illumina Hiseq200/2500 was used to sequence the constructed library, and the sequencing read length was 1×50 bp. After that, the samples were also sequenced on this platform.

Raw data by sequencing were submitted to an in-house program, ACGT101-miR (LC Sciences Houston, Texas, USA), to remove the 3’ connectors and junk sequences, and clean data were achieved. The clean data were mapped to databases of mRNA, Rfam and Repbase, and after filtering, valid data were obtained and used for miRNA identification. Subsequently, unique sequences of 18-26 nt in length were mapped to specific species precursors in miRbase 22.0 by BLAST searching, and known or novel miRNAs were annotated. After that, differentially expressed miRNAs (DE-miRNAs) between the plasma-derived exosomes of thin and normal Bactrian camels were identified using Student t test, and the thresholds for DE-miRNAs selection were |log2Fold change (FC)| > 1 and *P* value ≤ 0.05. Next, the potential target genes of the identified DE-miRNAs were predicted using the TargetScan and miRanda databases, and then were submitted to Gene Ontology (GO) and Kyoto Encyclopedia of Genes and Genomes (KEGG) pathway analyses. A *P* value ≤ 0.05 was set as the criterion of the significantly enriched GO terms and KEGG pathways.

### Proteomics analysis of exosomes

The exosomes from the plasma of thin and normal Bactrian camels (n = 5 for each group) were further sent to OBiO Technology (Shanghai) Corp., Ltd for proteomics analysis. Briefly, SDT lysate (4% SDS, 100mM Tris-HCl, pH 7.6) was added in the plasma-derived exosomes of thin and normal Bactrian camels, and then bathed in boiling water for 15min. After centrifuged at 14000 g for 15 min, the supernatant was collected, and then FASP ultrafiltration method was adopted to remove the SDT solution in the supernatant (28). Then, 100μg peptide of each sample was labeled using a TMT labeling kit (Thermo) according to the manufacturer’s protocols. The labeled peptides in each group were mixed, and graded by Agilent 1260 Infinity II HPLC system. Thereafter, each sample was separated using Easy nLC system, and then analyzed with a Q Exactive plus mass spectrometer.

The raw data generated by Q Exactive plus were converted to the.mgf format using Proteome Discoverer 2.2 (Thermo Fisher Scientific) software, and then submitted to MASCOT2.6 for protein annotation. Differentially expressed proteins (DEPs) between the plasma-derived exosomes of thin and normal Bactrian camels were screened based on the thresholds of FC > 1.2/FC < 0.85 and *P* < 0.05. Afterwards, the screened DEPs were subjected to GO terms, KEGG pathways and subcellular localization analyses.

### Cell culture and construction of MHCC-97H-LUC cells

A human hepatoma cell line MHCC-97H cells were purchased from Cell Bank, Chinese Academy of Sciences (Shanghai, China), and cultured in Dulbecco’s modified Eagle’s medium (DMEM, Thermo Fisher Scientific, Waltham, MA, USA) supplemented with 10% fetal bovine serum (FBS, Thermo Fisher Scientific). Then, the cells were maintained in an incubator with 5% carbon dioxide at 37°C. The MHCC-97H cells were passaged upon reaching 80%-90% confluence.

The MHCC-97H cells with LUC fluorescence (MHCC-97H-LUC cells) were constructed using lentivirus (H7656 pLenti-CBh-3xFLAG-Luc2-tCMV-mNeonGreen-F2A-Puro-WPRE, OBiO Technology (Shanghai) Corp., Ltd) package. Briefly, The MHCC-97H cells were seeded into a 6-well plate at a density of 3×10^5^ cells/well, and cultured overnight. After that, the cells were transfected with H7656 lentivirus, and then 10 μL polybrene (1 mg/mL) was added to each well at a final concentration of 5 μg/mL. After 24 h of transfection, the medium was changed to fresh medium, and after 72 of culture, final concentration of 2 μg/mL puromycin was added to select the stable transfection cell line (MHCC-97H-LUC cells).

### Animal experiments

A total of 21 SPF female Balb/c-nude mice aged 5-6 weeks were purchased from Jiangsu GemPharmatech Biotechnology Co. Ltd (Jiangsu, Chian). During the experiments, all the mice were free access to food and water, and maintained under controlled temperature (24 ± 2°C) and humidity (50 ± 10%) conditions, with a 12 h light/dark cycle. After acclimatization for 7 days, all the mice were randomly and equally divided into three groups as follow (n = 7 for each group): control group, N-Exo and T-Exo groups. All the mice were used to perform *in situ* tumor of the liver as previously described (29). Briefly, the mice were anesthetized using an isoflurane air hemp machine. After anesthesia, the liver lobe was gently extruded, and 40 μL MHCC-97H-LUC cell suspension (5×10^6^ cells/mouse) was injected to the liver. After stopping bleeding, the liver lobe was slowly returned to the abdominal cavity, sutured, and the wound was disinfected with iodophor. The animals were kept in a state of gas anesthesia all the time. After seven days, the mice in the N-Exo and T-Exo groups were respectively injected with 200 μg normal Bactrian camel plasma-derived exosomes and thin Bactrian camel plasma-derived exosomes through a tail vein once a week for three weeks (30, 31). The mice in the control group were treated with equal amount of PBS.

At the end of the experiment, all the mice were used for *in vivo* imaging using *in vivo* imaging system (PE LuminaLT series III, USA) (32). After that, the mice were sacrificed by cervical dislocation, and the liver tissues were collected for hematoxylin eosin (HE) staining to observe the effects of normal and thin Bactrian camel plasma-derived exosomes on tumor growth. All animal experiments were conducted in accordance with the National Medical Advisory Committee (NMAC) guidelines using approved procedures of the Experimental Animal Welfare and Ethics Committee of Inner Mongolia Agricultural University (No. NND2021094).

## Results

### Exosomes successfully isolated from the plasma of thin and normal Bactrian camels by ultra-centrifugation

**Figure 1:**
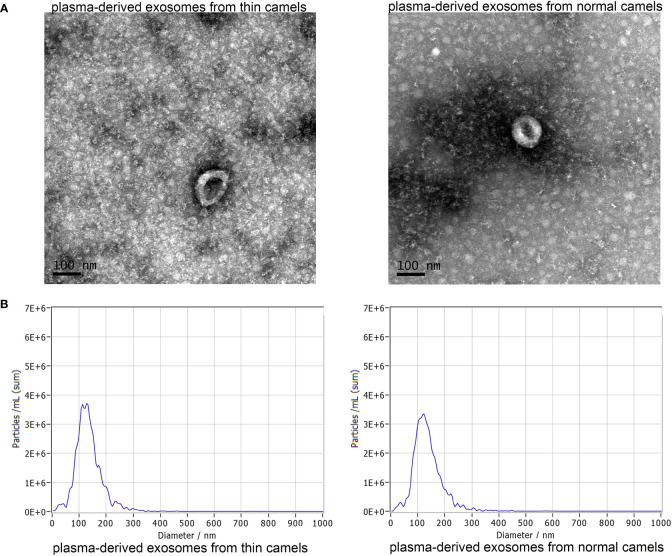
Characterization of exosomes from the plasma of thin Bactrian camels and normal Bactrian camels. **(A)** The morphology of the two exosomes visualized using a transmission electron microscopy. scale bar = 100 nm. **(B)** A Nano particle tracking analyzer used to measure the exosomes size distributions. Left: plasma-derived exosomes from thin camels; right: plasma-derived exosomes from normal camels.

Exosomes were respectively isolated from the plasma of thin and normal Bactrian camels, and then TEM and NTA were used to characterize them. TEM results showed that the exosomes isolated from the plasma of thin and normal Bactrian camels both exhibited a cup-shaped or round morphology with a diameter of approximately 100 nm (**Figure 1A**). Furthermore, NTA analysis indicated that the major peak in particle size of the exosomes from the plasma of thin and normal Bactrian camels were about 124.6 nm and 117.9 nm, respectively; and the overall size distribution ranged from 50 nm to 200 nm (**Figure 1B**). These were in line with the size distribution of exosomes previously reported (33, 34). All the results implied that the exosomes were successfully extracted from the plasma of thin and normal Bactrian camels using the ultra-centrifuge method.

### Effects of normal and thin Bactrian camel plasma-derived exosomes on tumor growth

**Figure 2:**
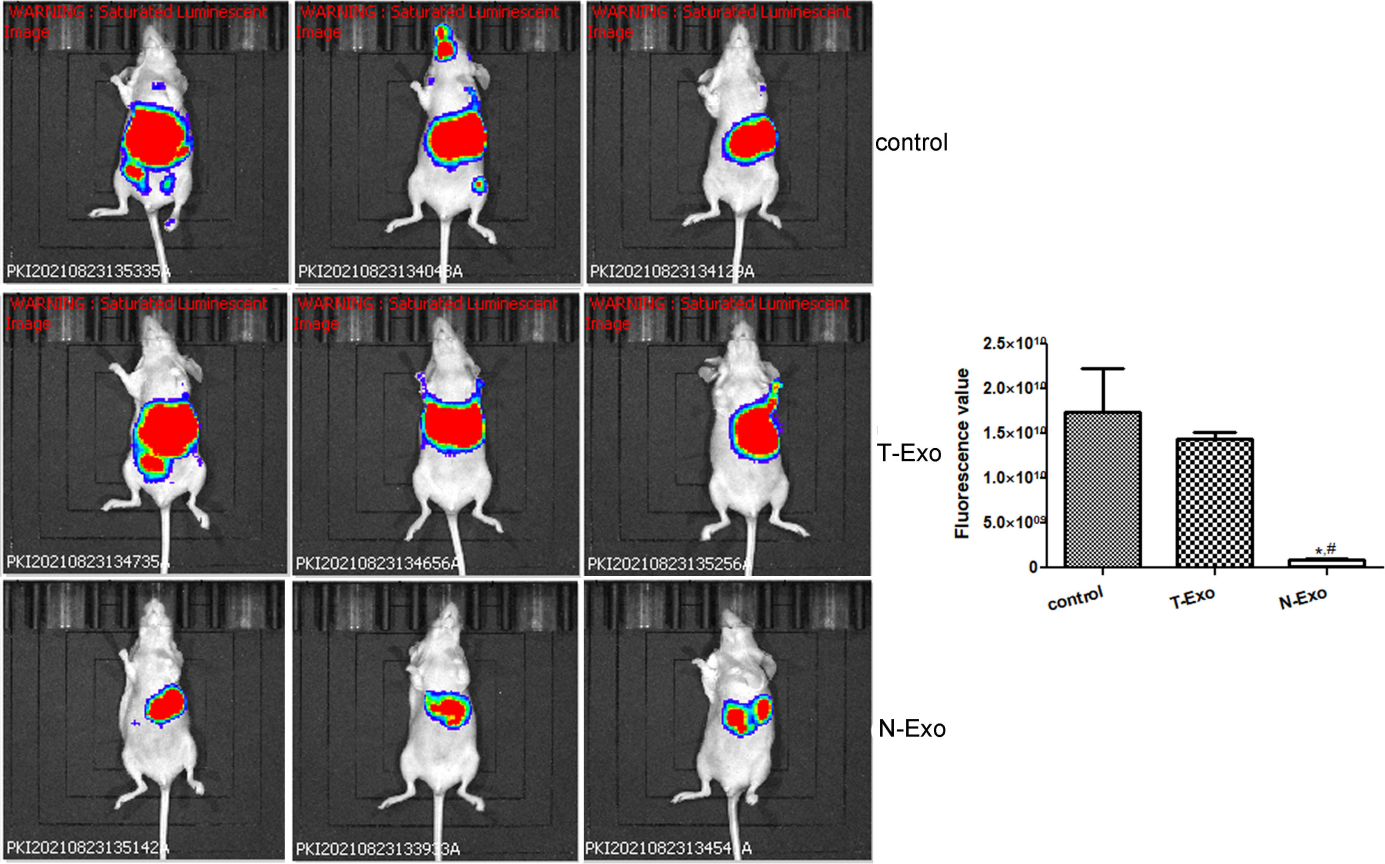
Representative images of *in vivo* imaging and quantitative analysis in the mice with different treatments. *: *P* < 0.05, compared with the control group; #: *P* < 0.05, compared with the T-Exo group. T-Exo: plasma-derived exosomes from thin camels; N-Exo: plasma-derived exosomes from normal camels.

**Figure 3:**
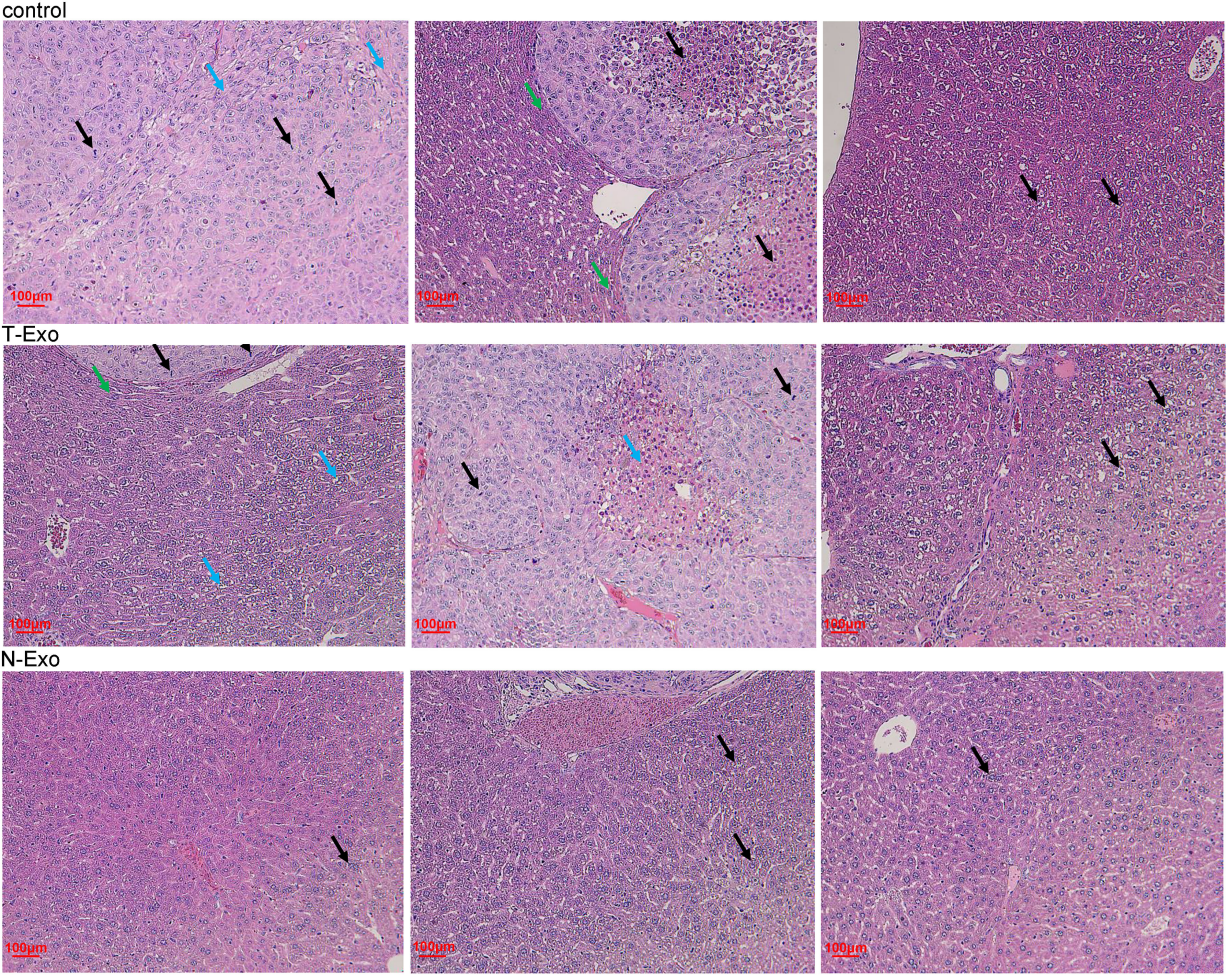
Representative pictures of hematoxylin eosin staining of liver tissues in the mice with different treatments. Black arrow: hepatic cells; blue arrow: fibrous hyperplasia; green arrow: peripheral hepatocyte cords. T-Exo: plasma-derived exosomes from thin camels; N-Exo: plasma-derived exosomes from normal camels.

In order to understand the function of Bactrian camel plasma-derived exosomes on liver tumor growth, *in vivo* imaging of the mice was performed. After imaging, there was no significant difference in the fluorescence value between the control mice and the mice treated with thin Bactrian camel plasma-derived exosomes (*P* > 0.05); but the fluorescence value in the mice treated with normal Bactrian camel plasma-derived exosomes was significantly decreased compared to the control mice and the mice with thin Bactrian camel plasma-derived exosomes (*P* < 0.05, **Figure 2**). Additionally, HE staining of liver tissues showed that in the control mice, the normal hepatic nodule structure disappeared, the tumor cells showed clumpy and solid growth, peripheral hepatocyte cords were compressed and atrophied (green arrow); as well as more mitotic phases (black arrow) and slight fibrous hyperplasia were seen. The morphology of liver tissues in the mice with thin Bactrian camel plasma-derived exosomes was similar with that in the control mice (**Figure 3**). However, in the mice with normal Bactrian camel plasma-derived exosomes, hepatic lobule structure was clear, hepatocyte cords were arranged neatly, hepatic sinuses were obvious, a few hepatic cells were slightly vacuolated (black arrow), and no obvious inflammatory cell infiltration and tumor cells were observed (**Figure 3**). All these results indicated that compared with the thin Bactrian camel plasma-associated exosomes, normal Bactrian camel plasma-derived exosomes could inhibit liver tumor growth, which may have potential therapeutic effects on HCC.

### Identification of DE-miRNAs between the plasma-derived exosomes from thin and normal Bactrian camels, and functional analysis

**Figure 4:**
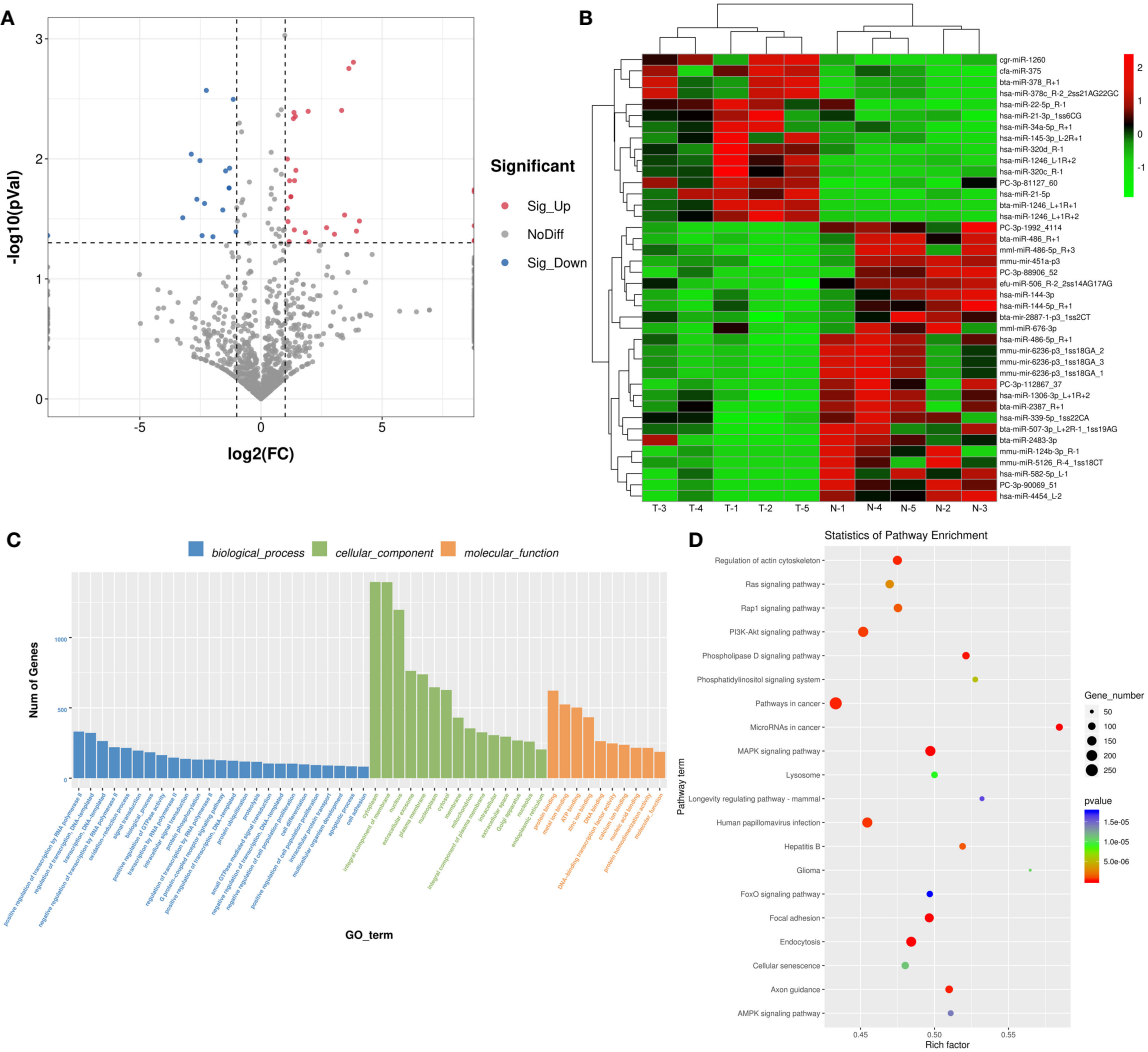
Identification of differential expressed microRNAs (DE-miRNAs) between the plasma-derived exosomes from thin and normal Bactrian camels. **(A)** The volcano figure of DE-miRNAs. **(B)** The bidirectional hierarchical cluster analysis of these DE-miRNAs. **(C)** Gene Ontology (GO) terms of these DE-miRNAs in biological process, cellular component, and molecular function. **(D)** Kyoto Encyclopedia of Genes and Genomes (KEGG) pathways enrichment of these DE-miRNAs.

Due to the different effects of normal and thin Bactrian camel plasma-associated exosomes on tumor growth, we further investigated the differences of the two kinds of exosomes using small RNA sequencing and proteomics. After sequencing and analyzing, a total of 40 DE-miRNAs were identified between the exosomes from the thin and normal Bactrian camels based on the criteria of |log_2_FC| > 1 and *P* < 0.05, including 15 down-regulated and 25 up-regulated DE-miRNAs in the normal Bactrian camel plasma-derived exosomes (**Figure 4A** and **Supplementary Table 1**). The bidirectional hierarchical clustering heatmap of these DE-miRNAs showed that all the identified DE-miRNAs could well distinguish the thin Bactrian camel plasma-derived exosomes from the normal Bactrian camel plasma-derived exosomes (**Figure 4B**).

After that, 22602 target genes of these DE-miRNAs were predicted using the TargetScan and miRanda databases, and then were significantly enriched in 626 GO terms and 149 KEGG pathways. As shown in **Figure 4C**, these DE-miRNAs were significantly related to “positive regulation of transcription by RNA polymerase II”, “regulation of transcription, DNA-template” and “oxidation-reduction process” in biological process (BP) GO terms; and “cytoplasm”, “integral component of membrane”, and “nucleus” in cellular component (CC) GO terms; as well as “protein binding”, “metal binding” and “ATP binding” in molecular function (MF) GO terms. Additionally, the significantly enriched KEGG pathways of these DE-miRNAs included “Ras signaling pathway”, “Rap1 signaling pathway”, “PI3K-Akt signaling pathway”, “MAPK signaling pathway”, “AMPK signaling pathway”, “FoxO signaling pathway”, “phospholipase D signaling pathway” and “phosphatidylinositol signaling system” (**Figure 4D**).

### Screen of DEPs between the plasma-derived exosomes from thin and normal Bactrian camels

**Figure 5:**
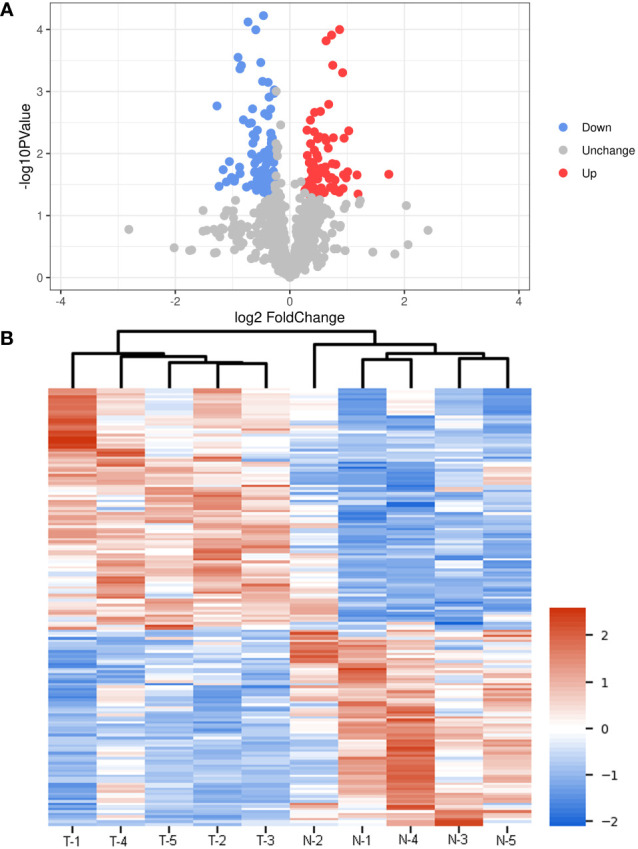
Screen of different expressed proteins (DEPs) between the plasma-derived exosomes from thin and normal Bactrian camels. **(A)** The volcano figure of DEPs. **(B)** The heatmap of these screened DEPs in the different samples.

According to the thresholds of FC > 1.2/FC < 0.85 and *P* < 0.05, 172 DEPs were screened out, including 77 up-regulated (such as angiogenin, apolipoprotein A-II, cornulin, kinase suppressor of Ras 1, hepatocyte growth factor activator, Ras-related protein Rab-11B, and integrin-linked protein kinase) and 95 down-regulated (complement C1q subcomponent subunit B, phosphoserine aminotransferase, CD5 antigen-like, and claudin-5) DEPs in the normal Bactrian camel plasma-associated exosomes compared with the thin Bactrian camel plasma-derived exosomes (**Figure 5A** and **Supplementary Table 2**). The heatmap of these DEPs between the plasma-derived exosomes from thin and normal Bactrian camels was shown in **Figure 5B**, which indicated that these screened DEPs could differentiate the two kinds of exosomes well.

### Functional and subcellular localization analyses of these DEPs

**Figure 6:**
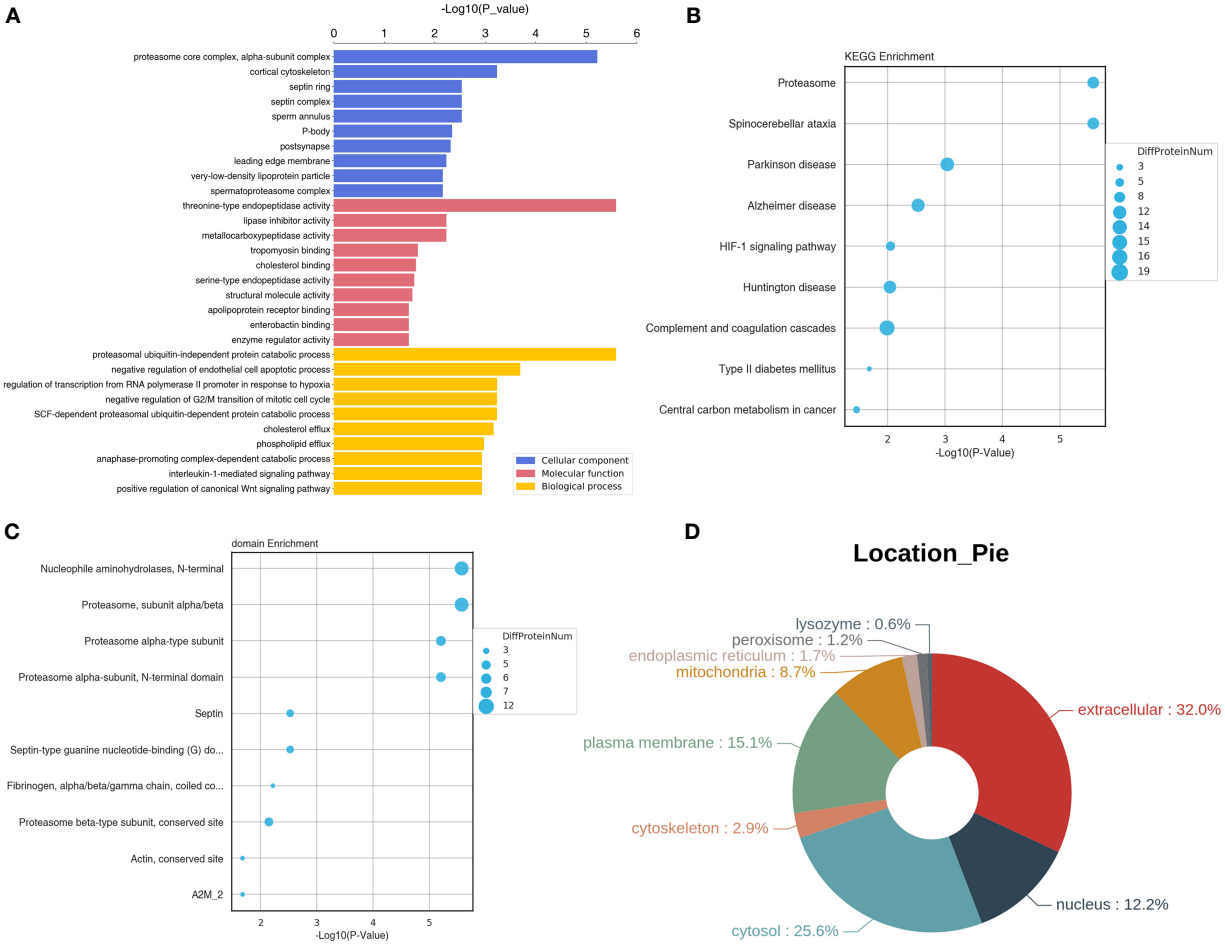
Functional and subcellular localization analyses of the identified DEPs. **(A)** GO terms of the identified DEPs. **(B)** Top 10 KEGG pathway enrichment of these DEPs. **(C)** Top 10 significant enriched protein domains of these DEPs. **(D)** The Pie of subcellular localization of the screened DEPs.

These DEPs were further submitted for GO terms, KEGG pathways and subcellular location analyses. It is clear that these DEPs were significantly enriched in “proteasome core complex, alpha-subunit complex”, “cortical cytoskeleton”, “septin ring” and “sperm annulus” of CC GO terms, and “threonine-type endopeptidase activity”, “lipase inhibitor activity”, and “metallocarboxypeptidase activity” of MF GO terms, and “proteasomal ubiquitin-independent protein catabolic process”, “negative regulation of endothelial cell apoptotic process”, “negative regulation of G2/M transition of mitotic cell cycle”, “interleukin-1-mediated signaling pathway” and “positive regulation of canonical Wnt signaling pathway” of BP GO terms (**Figure 6A**). KEGG enrichment analysis showed that these DEPs were related to “HIF-1 signaling pathway”, “complement and coagulation cascades”, and “central carbon metabolism in cancer” (**Figure 6B**).

In addition, these DEPs were significantly enriched in some protein domains, such as “nucleophile aminohydrolases, N-terminal”, “proteasome, subunit alpha/beta”, “septin”, “actin, conserved site” and “fibrinogen, alpha/beta/gamma chain, coiled conserved site” (**Figure 6C**). Finally, subcellular location analysis found that the most DEPs located in extracellular (32%), followed by cytosol (25.6%), plasma membrane (15.1%), nucleus (12.2%), and mitochondria (8.7%). A small number of the DEPs were in cytoskeleton (2.9%), endoplasmic reticulum (1.7%), peroxisome (1.2%) and lysozyme (0.6%) (**Figure 6D**).

## Discussion

HCC is highly invasive, fast-growing and insidious onset, which poses a serious threat to people’s health and life (35). Exosomes, released by various cells, are reported to participate in the occurrence and development of HCC (36), and previous studies have elaborated that camel milk-derived exosomes have an inhibitory effect on breast (MCF7 cells) and liver cancer (HepaRG cells) cells, thus acting as anticancer adjuvant agents (12, 13). However, the roles of different camel plasma-associated exosomes in HCC remain unclear. This study was the first time to investigate the effects of exosomes isolated from the plasma of Bactrian camels of different body sizes on HCC, and further explore their differences at transcriptome and proteomics levels. In this study, exosomes were isolated from the plasma of thin and normal Bactrian camels. Due to the lack of the corresponding antibodies, TEM and NTA were only used to characterize the isolated exosomes. It was found that the morphology of the isolated exosomes was cup-shaped of nearly round, and the major peak of the exosomes was about 120 nm, which were quite similar with the results of human/mice plasma-derived exosomes (37–39). Therefore, the exosomes were successfully isolated from Bactrian camel plasma through this ultracentrifugation method, and can be used for subsequent experiments.

In order to determine the effects of the plasma-derived exosomes from thin and normal Bactrian camels on HCC tumor growth, MHCC-97H cells were injected to the liver of the nude mice, and the two exosomes were then treated with the mice. MHCC-97H cells are a kind of highly metastatic liver HCC cell lines, and are often used to induce orthotopic liver tumors by injection into nude mice. In a previous study of Zhou et al., MHCC-97H cell suspension was injected to the same part of the left liver lobe of nude mice to form *in situ* liver tumors, and *in vivo* tumorigenesis experiments were conducted, as well as it was found that *ZEB1* knockout in MHCC-97H cells significantly reduced the weight and size of primary tumors and the number of metastases on the liver surface (40). Another study created a tumor mouse model by subcutaneously injecting MHCC-97H cell suspension transfected with miR-30a-3p or miRNA negative controls into the right side of female nude mice, and then the roles of miR-30a-3p in HCC were further investigated (41). Our study also used MHCC-97H cell suspension to perform *in situ* tumor of the liver, and after modeling, different exosomes from the plasma of Bactrian camels were treated the mice. *In vivo* imaging and HE staining showed that no significant differences were found in fluorescence value and liver tissue morphology between the control mice and the mice treated with the exosomes from thin Bactrian camels; while the fluorescence value and the live histology changes were alleviated in the mice with the exosomes from normal Bactrian camels. Zhu et al. (42) showed that natural killer cells-derived exosomes treatment could evidently decrease the signal intensity after melanoma xenograft compared with the control, which indicated the immunotherapeutic capacity of the natural killer cells-derived exosomes in melanoma. Another study also used *in vivo* imaging and found that DiR fluorescence was stronger after intracerebroventricularly administrated with human adipose mesenchymal stem cell-derived exosomes (hADSC-ex), which suggested that exosomes were accumulated in rat brain, and combined with the physiological indicators, hADSC-ex could promote sensorimotor functional recovery in a traumatic brain injury rat model (43). Taken together, we concluded that normal Bactrian camel plasma-associated exosomes could suppress the growth of live cancer cells (MHCC-97H) and relieve liver tissue pathological changes, so they may have potential immunotherapeutic roles in HCC. These results suggest that normal Bactrian camel plasma-derived exosomes may be potential for HCC treatment and management.

Exosomes can effectively deliver drugs, miRNA or proteins to specific cells or tissues, such as the intestine, colon, or liver, and may thereby be used as potential tools for the treatment cancer or other diseases (44). In addition to treating diseases, another important application of exosomes is that they can serve as biomarkers for disease diagnosis and prognosis (9). For example, the level of plasma-derived exosomal PD-L1 was strongly correlated with the progression of head and neck cancers (9); as well as elevated levels of miR-191, miR-21, and miR-451a in serum exosomes appeared to be biomarkers for pancreatic cancer (45). In our research, due to the different actions of normal and thin Bactrian camel plasma-associated exosomes in liver tumor growth, we further compared the differences of the two exosomes from different perspectives. After small RNA sequencing and proteomics analysis, 40 DE-miRNAs (15 down-regulated: hsa-miR-21-5p, hsa-miR-320c, hsa-144-3p/5p; and 25 up-regulated: hsa-miR-4454, hsa-miR-582-5p) and 172 DEPs (77 up-regulated: angiogenin, Ras-related protein Rab-11B, kinase suppressor of Ras 1; and 95 down-regulated: complement C1q subcomponent subunit B, claudin-5) were identified in the plasma-derived exosomes from normal Bactrian camels. A previous study of Pu et al. (46) demonstrated that extracellular vesicle-related hsa-miR-21-5p and hsa-144-3p were significantly elevated in the serum of HCC patients, which indicated the two miRNAs may be essential for HCC pathogenesis. Besides, a meta-analysis of seventeen animal and human studies reported that hsa-miR-320c was up-regulated after bariatric surgery (47), which was in accordance with our results. After comparing the thin and obese individuals, hsa-miR-4454 was consistently higher in obesity (48); whereas hsa-miR-582-5p was down-regulated in the visceral adipose tissues-derived exosomes of obese patients (49). They were found to be associated with diabetes and multiple cancer (50, 51). In addition, angiogenin is a stress-induced secreted ribonuclease with nuclear and cellular solute activities, and involved in normal development and disease (52). Ras-related protein Rab-11B is a key regulator of intracellular transport and endocytic pathway (53), and Rab proteins play important roles in HCC progression (54). Kinase suppressor of Ras 1 has been reported to attenuate neurostatin secretion and extracellular signal-regulated kinase 1 and 2 signaling in human endocrine cells, thereby participating in obesity-related metabolic disorders and Ras-driven cancer (55). Complement C1q subcomponent subunit B is involved in complement and coagulation cascades; and claudin-5 is an abundant tight junction protein at the blood-brain barrier, and its dysregulation is closely related to neurodegenerative disorders (56). These findings, together with our results, we speculate that the identified DE-miRNA (such as hsa-miR-21-5p, hsa-miR-320c, hsa-144-3p/5p, hsa-miR-4454, hsa-miR-582-5p) and DEPs (such as angiogenin, Ras-related protein Rab-11B, kinase suppressor of Ras 1, complement C1q subcomponent subunit B, claudin-5) may be involved in the development of HCC. However, the potential roles of these DE-miRNAs and DEPs in the pathogenesis of HCC deserve further investigation.

Further, these identified DE-miRNAs and DEPs were subjected to functional analysis, and found these DE-miRNAs and DEPs were significantly enriched in many signaling pathways, including Ras, Rap1, PI3K-Akt, MAPK, AMPK, FoxO, HIF-1, interleukin-1-mediated signaling pathway, and positive regulation of canonical Wnt signaling pathway. Ras signaling pathway plays a key role in cancer initiation and cell proliferation, and MAPK signaling pathway is the downstream of Ras, and can be regulated by Ras signaling (57). Rap1, a small GTP enzyme very similar to Ras, has been shown to combat and mimic Ras-driven cancer phenotypes (58). The presence of Rap1 in the nanoclusters can reduce the number of Ras molecules, thereby inhibiting Raf-1 activation and MAPK signaling (58). A previous study reported that imbalance of Ras/MAPK signaling is associated with HCC progression and prognosis, and Ras/MAPK pathway effectors can be considered as potential therapeutic targets in the HCC field (59). Many studies have demonstrated that PI3K/Akt pathway is activated in 40-50% of HCC patients and plays a critical role in cell growth and metabolism, ultimately affecting the invasion, metastasis, and invasiveness of cancer cells (60, 61). FoxO is negatively regulated by PI3K/Akt signaling pathway, and is believed to inhibit cell proliferation and induce cell cycle arrest and cell apoptosis (62). Hypoxia and inflammation are two key factors that shape the HCC microenvironment. The steady increase of HIF-1 can induce tumor-associated macrophages to secrete more interleukin-1β in response to hypoxia, thus promoting epithelial mesenchymal transition and metastasis of HCC cells (63). Zhu et al. (64) found that TRIM24 knockdown inhibited proliferation and migration of HCC cells, and AMPK knockdown mitigated the action of TRIM24 knockdown in HCC cells, which suggested that TRIM24 could accelerate HCC development *via* AMPK signaling. Additionally, canonical Wnt signaling pathway is involved in the activation of β-catenin, and the abnormal activation of Wnt/β-catenin signaling contributes to the occurrence and progression of liver cancers, including HCC and cholangiocarcinoma (65). Combined with our results, it can be inferred that normal Bactrian camel plasma-derived exosomes may suppress tumor growth of HCC through Ras, Rap1, PI3K-Akt, MAPK, AMPK, FoxO, HIF-1, interleukin-1, and canonical Wnt signaling pathways. However, how normal Bactrian camel plasma-derived exosomes play specific roles in HCC through these pathways remains to be further studied.

However, there are some limitations in our research. Firstly, the potential roles of these DE-miRNAs and DEPs in the pathogenesis of HCC deserve further investigation, as well as how normal Bactrian camel plasma-derived exosomes play specific roles in HCC through the enriched pathways remains to be further studied. Secondly, UTR assay or other functional studies should be carried out to functionally validate the identified DE-miRNAs, and their target genes, as well as to confirm their association. Additionally, in the future, male mice and clinical trials were also needed to be used for further verification of our findings.

## Conclusion

In conclusion, normal Bactrian camel plasma-derived exosomes could inhibit the growth of HCC cells and tumor, thus having potential therapeutic effects on HCC. After comparing the exosomes from the plasma of thin and normal Bactrian camels, it was found that the identified 40 DE-miRNAs and 172 DEPs may be involved in the HCC progression, and normal Bactrian camel plasma-derived exosomes may repress tumor growth of HCC by Ras, Rap1, PI3K-Akt, MAPK, AMPK, FoxO, HIF-1, interleukin-1, and canonical Wnt signaling pathways. These findings provide evidence for normal Bactrian camel plasma-derived exosomes in the clinical treatment of HCC, and lay the foundation for the screened DE-miRNAs, DEPs and enriched signaling pathways as novel targets and pathways for HCC therapy in clinical settings.

## Data availability statement

The datasets presented in this study can be found in online repositories. The names of the repository/repositories and accession number(s) can be found in the article/**Supplementary Material**.

## Ethics statement

The animal study was reviewed and approved by The Experimental Animal Welfare and Ethics Committee of Inner Mongolia Agricultural University (No. NND2021094).

## Author contributions

HY and SY conceived and guided the whole project. HY, SY and XW conducted the sample collection. HY, YL, MW, SX, YC and ZX performed the experimental procedures and statistical analysis. SY, YL and XW organized the database. HY and SY wrote and the manuscript text. All authors contributed to the article and approved the submitted version.
