# Supplementary material for: Effects of plasma-derived exosomes from the normal and thin Bactrian camels on hepatocellular carcinoma and their differences at transcriptome and proteomics levels
Source: Front Oncol. 2023 Feb 2;13:994340. doi: 10.3389/fonc.2023.994340 (PMC9933125; doi:10.3389/fonc.2023.994340)
Supplement: Supplementary file 1 [file DataSheet_1.zip › Supplementary Material/Supplementary Tables Caption.docx]

**Supplementary Table 1.** All differentially expressed miRNAs (DE-miRNAs) between the plasma-derived exosomes from thin and normal Bactrian camels with the thresholds of |log2Fold Change (FC)| > 1 and *P* < 0.05.

**Supplementary Table 2.** All differentially expressed proteins (DEPs) between the plasma-derived exosomes from thin and normal Bactrian camels with the thresholds of FC > 1.2/FC < 0.85 and *P* < 0.05.
